# Medical Emergencies at the Royal Edinburgh Hospital (May 2018–January 2025)

**DOI:** 10.1192/bjo.2026.11469

**Published:** 2026-06-30

**Authors:** Naheed Raza, Brenda Binnie, Joseph Mearns, Chris O'Shea, Evonne Rendall

**Affiliations:** NHS Lothian, Edinburgh, United Kingdom

## Abstract

**Aims:**

To understand the frequency, nature and location of medical emergencies at the Royal Edinburgh Hospital, (REH) a stand-alone psychiatric institute, between May 2018 and January 2025.

Resident doctors at the Royal Edinburgh Hospital are part of a small crash team responding to medical emergencies across the Royal Edinburgh Hospital, a stand-alone psychiatric facility in Edinburgh with 29 wards and approximately 570 beds. Inpatient units included acute adult wards, an intensive psychiatric care unit, rehabilitation and psychiatry of old agewards. Additionally, there are specialist day services for young patients, a medium secure forensic unit, residential unit for learning disability patients and an acquired brain injury unit.

As a non-acute site, access to emergency medications and resources is limited. Additionally the site is large, complex and difficult to navigate. Knowing what medical emergencies occur and where could help resident doctors prepare for their on-calls and improve the patient outcomes.

**Methods:**

2222 medical emergency calls made between May 2018 and January 2025 were reviewed by checking yellow forms completed after a medical emergency and collecting information from switchboard.

**Results:**

The frequency of medical emergencies increased between May 2018 and January 2025 with an average of 5–6 calls per month by January 2025.

The most common 2222 call sites were the acute adult wards, followed by psychiatry of old age wards and the rehabilitation wards.

The most common cause of an emergency was seizure (20.9%), followed by choking (11.8%), ligature (10.6%), overdose (6.9%) and unresponsive episodes (6.5%).

**Conclusion:**

Little published research appears to exist on medical emergencies at stand-alone psychiatric hospitals.

These findings have been shared locally and resulted in multiple interventions, for example the creation of a new seizure protocol which better reflects the skills and background of the responding team, a new trainee site map, amended simulation training which is tailored to staff working in different areas, new trainee induction talk and 2222 focused site tour for rotating doctors.

New resident doctors have reported increased confidence in approaching their on-calls and attending medical emergencies as a result of these interventions.

